# EGFR Signaling Regulates Maspin/SerpinB5 Phosphorylation and Nuclear Localization in Mammary Epithelial Cells

**DOI:** 10.1371/journal.pone.0159856

**Published:** 2016-07-22

**Authors:** Mariana Tamazato Longhi, Magna Magalhães, Jeffrey Reina, Vanessa Morais Freitas, Nathalie Cella

**Affiliations:** Departamento de Biologia Celular e do Desenvolvimento, Instituto de Ciências Biomédicas, Universidade de São Paulo, São Paulo, SP, Brazil; University of Munich, GERMANY

## Abstract

Maspin (SerpinB5) is a non-inhibitory serpin (serine protease inhibitor) with very diverse biological activities including regulation of cell adhesion, migration, death, control of gene expression and oxidative stress response. Initially described as a tumor and metastasis suppressor, clinical data brought controversies to the field, as some studies reported no correlation between SerpinB5 expression and prognosis value. These data underscore the importance of understanding SerpinB5 function in a normal physiological context and the molecular mechanism involved. Several SerpinB5 phosphoforms have been detected in different cell lines, but the signaling pathways involved and the biological significance of this post-translational modification *in vivo* remains to be explored. In this study we investigated SerpinB5 expression, subcellular localization and phosphorylation in different stages of the mouse mammary gland development and the signaling pathway involved. Here we show that SerpinB5 is first detected in late pregnancy, reaches its highest levels in lactation and remains at constant levels during post-lactational regression (involution). Using high resolution isoelectric focusing followed but immunoblot, we found at least 8 different phosphoforms of SerpinB5 during lactation, which decreases steadily at the onset of involution. In order to investigate the signaling pathway involved in SerpinB5 phosphorylation, we took advantage of the non-transformed MCF-10A model system, as we have previously observed SerpinB5 phosphorylation in these cells. We detected basal levels of SerpinB5 phosphorylation in serum- and growth factor-starved cells, which is due to amphiregulin autocrine activity on MCF-10A cells. EGF and TGF alpha, two other EGFR ligands, promote important SerpinB5 phosphorylation. Interestingly, EGF treatment is followed by SerpinB5 nuclear accumulation. Altogether, these data indicate that SerpinB5 expression and phosphorylation are developmentally regulated. *In vitro* analyses indicate that SerpinB5 phosphorylation is regulated by EGFR ligands, but EGF appears to be the only able to induce SerpinB5 nuclear localization.

## Introduction

Maspin (SerpinB5) is a non inhibitory serpin (serine protease inhibitor) with very diverse biological activities, including increase in cell adhesion, inhibition of cell migration, control of gene transcription, modulation of apoptosis and oxidative stress response. First described in the mammary tissue, it is now clear that SerpinB5 is expressed by most epithelial cells. *In vitro* and animal studies indicated SerpinB5 has an important tumor and metastasis suppressor activity, which was mainly associated with its effects on adhesion, migration, cell death and angiogenesis inhibition. Clinical data, however, brought controversies to the field: whereas some studies found a correlation between loss of SerpinB5 and tumor progression in different cancer types including breast [1–3], prostate [4], lung [5, 6] and skin [7], others observed an opposite trend [8–12]. Another unresolved issue is how SerpinB5 subcellular localization is related to its tumor suppressor activity, as nuclear SerpinB5 is associated with a good prognostic in some cases [13–17], but not in others [18, 19]. These observations may indicate that SerpinB5 biological activities are cell type and tissue context-dependent. The fact that most studies have been conducted in cell lines or in pathological conditions may also account for these divergences. These data underscore the importance of understanding how SerpinB5 is regulated in a non-transformed model as well as in a physiological context. SerpinB5 phosphorylation has been identified in mammary and corneal epithelial cell lines [20–22]. Whether this modification occurs *in vivo* and the signaling pathways involved remain to be elucidated. In this study we characterized SerpinB5 expression, subcellular localization and phosphorylation in different stages of the mouse mammary gland development and investigated the underlying signaling pathway in the non-transformed MCF-10A model system, which expresses SerpinB5 endogenously. Here we show that SerpinB5 protein is detected from late pregnancy, throughout lactation and involution and it is predominantly detected in the cytoplasm of mammary epithelial cells. Several SerpinB5 phosphoforms were detected in the lactating gland, but not in other developmental stages. In MCF-10A cells, phosphorylated SerpinB5 is detected even in serum and growth-factor starved cells, but increases significantly upon EGF (epidermal growth factor) and TGF alpha (transforming growth factor alpha) treatment. Interestingly, we observed that EGF-treatment is accompanied by an increase in SerpinB5 nuclear localization. Altogether, these data indicate that SerpinB5 expression and phosphorylation is developmentally regulated and EGFR (epidermal growth factor receptor) signaling regulates SerpinB5 phosphorylation and nuclear localization.

## Material & Methods

### Cell Culture

The MCF-10A cell line was obtained from Banco de Células do Rio de Janeiro, Rio de Janeiro–RJ, Brazil. Cells were grown in DMEM/F12 medium supplemented with antibiotics, EGF (10 ng/ml), insulin (10 ug/ml), cholera toxin (1 ug/ml), hydrocortisone (1 ug/ml) and heat-inactivated horse serum (5%). Soluble factors were from Sigma (EGF, insulin, cholera toxin, hydrocortisone) or Gibco (TGF alpha)., When necessary, 1 ug/ml of goat anti-amphiregulin (AREG)-neutralizing antibody (R&D AB-262-NA) or control goat IgG (eBiosciences) were added to starved cells for 24 hours. Recombinant SerpinB5 was from Sigma.

### Mouse maintenance and mammary gland processing

Female Balb/c mice were obtained from the animal facility of Instituto de Ciências Biomédicas of Universidade de São Paulo. They were housed in a light and temperature–controlled room (12 h light/dark cycle, 21±2°C) and provided with food and water *ad libitum*. For isolation of mammary tissue from different developmental stages, animals were mated and the vaginal plug was monitored to ensure day 1 of pregnancy and so on. Day 1 of involution was considered the day after the 21^st^ day of lactation, when pups were weaned. When necessary, mice were placed in an euthanasia chamber. Carbon dioxide was injected from a tank into the chamber at appropriate flow rate. Animals were left in the chamber until clinical death was ensured. This study was carried out in strict accordance with the guidelines of the Animal Ethics Committee of Instituto de Ciências Biomédicas of Universidade de São Paulo. The protocol was approved by this Committee (Permit Number: 161). All efforts were made to minimize suffering.

### 2D-SDS-PAGE

Protein samples (1.7–2 mg) were applied onto 17 cm 4.7–5.9 (17 cm) linear immobilized pH gradient strips (Bio-Rad ReadyStrip™ IPG strip) in rehydration buffer (8M urea, 4% CHAPS, 40 mM DTT) for 16 h at room temperature. Isoelectric focusing (IEF) was performed on IPGphor III apparatus (GE Healthcare) at 17 kV h. For the second dimension, strips were incubated at room temperature for 20 min in equilibration buffer (6 M urea, 2% SDS, 50 mM Tris–HCl, pH 6.8, 30% glycerol and 0.001% bromophenol blue with 2% DTT), followed by incubation with 4% iodoacetamide in equilibrium buffer for 20 min. The second dimension was performed in a vertical 12% SDS-PAGE. Proteins were transferred to PVDF membrane and analyzed by Western blot with anti-SerpinB5 antibodies (see below).

#### Protein extraction, subcellular fractionation, Phos-Tag™ SDS-PAGE and Western Blot

Inguinal glands were dissected from virgin animals or at specific developmental time-points during pregnancy, lactation and involution. Tissue was flash frozen and stored at -80°C. Mammary epithelial nuclear and cytoplasmic fractions were prepared with the NER-PER™ Nuclear and Cytoplasmic Extraction Reagent Kit (Thermo Fisher) according to manufacturer’s instructions. In order to prepare protein extracts compatible with IEF analysis, frozen tissues were grounded with a chilled mortar and pestle in liquid nitrogen and solubilized in 2-D Protein Extraction Buffer 1 (GE Healthcare cat# 28-9435-22) with the help of the Sample Grinding Kit (GE Healthcare cat# 80-6483-37). Extracts were cleared by centrifugation and quantified by the Bradford method (Bio-Rad). 1.7–2 mg of protein extracts were precipitated in 3 volumes of acetone at -20°C for at least 2 h and ressuspended in rehydration buffer. Phos-Tag™ SDS-PAGE gels were prepared following manual instructions and published protocol (Wako Pure Chemical Industries Ltd.) [23]. Protein extracts (from tissue or MCF-10A cells) for regular SDS-PAGE or Phos-Tag™ SDS-PAGE were prepared in RIPA buffer [24]. 50–100 ug of extracts were separated by 12% SDS-PAGE or by 8% SDS-PAGE Phos-Tag™ gels containing 50 um of Phos-Tag™-conjugated acrylamide to separate the phosphorylated species. Proteins were transferred to PVDF membrane and probed with diverse antibodies as follows: mouse monoclonal anti-SerpinB5 (Millipore cat# 4035) 1:10,000, mouse monoclonal anti-SerpinB5 (BD Pharmingen cat# 554292) 1:5,000, rabbit anti-SerpinB5 (Sigma *Prestige Antibodies*® HPA019132) 1:10,000, rabbit anti-SerpinB5 (Santa Cruz sc-22762) 1:1,000, anti-alpha tubulin (Sigma) 1:2,000, anti-HSP90 (Cell Signaling) 1:1,000 and anti-lamin B1 (ABCAM ab16048) 1:1,000.

### Mammary gland processing and immunofluorescence

Inguinal glands were dissected from lactating female Balb/c mice. Tissue was fixed in 4% paraformaldehyde overnight at 4°C before processing to paraffin blocks. For immunostaining, sections were deparaffinized, rehydrated, and subjected to 10 mM citrate buffer pH 6 antigen retrieval for 30 min at 95°C. MCF-10A cells were fixed with 2% paraformaldehyde in PBS for 20 min at room temperature. The fixed cells were permeabilized with 0.05% Triton X-100 in blocking buffer (5% goat serum in PBS) for 1 h at room temperature. Mammary gland tissue section and MCF-10A staining were performed with rabbit anti-SerpinB5 antibody (Sigma *Prestige Antibodies*® HPA019132 and HPA019136, respectively) 1:200, overnight at 4°C. All stained samples were mounted in Prolong Gold antifade reagent with DAPI (Molecular Probes). Secondary antibodies were goat anti-rabbit IgG (H+L) Alexa Fluor®568 or goat anti-rabbit IgG Alexa Fluor®488 F(ab’)2 Fragment (Molecular Probes).

### Microscope image acquisition

MCF-10A cells images were obtained with an Axiophot widefield fluorescence microscope using a 63x PlanApo 1.3 NA objective (Carl Zeiss). Images were acquired using a digital CCD monochromatic camera (CoolSnap HQ2, Photometrics Inc, Tucson, AZ, USA). The microscope and all devices were controlled by Metamorph Premier 7.6 software (Molecular Devices, Sunnyvale, CA, USA). For tissue, confocal images were obtained using a Zeiss LSM 780 system (Carl Zeiss, Jena, Germany) at Core Facilities to Support Research (CEFAP) at Sao Paulo University. Images acquired with a Plan-Apochromat 63x 1.4 NA oil immersion objective were rendered with Zen Software (Carl Zeiss, Jena, Germany). Photoshop CS3 (Adobe) was used when necessary to adjust levels within each channel equally across all images to maximize image clarity.

## Results

### SerpinB5 expression and phosphorylation during the development of the mouse mammary gland

**Fig 1:**
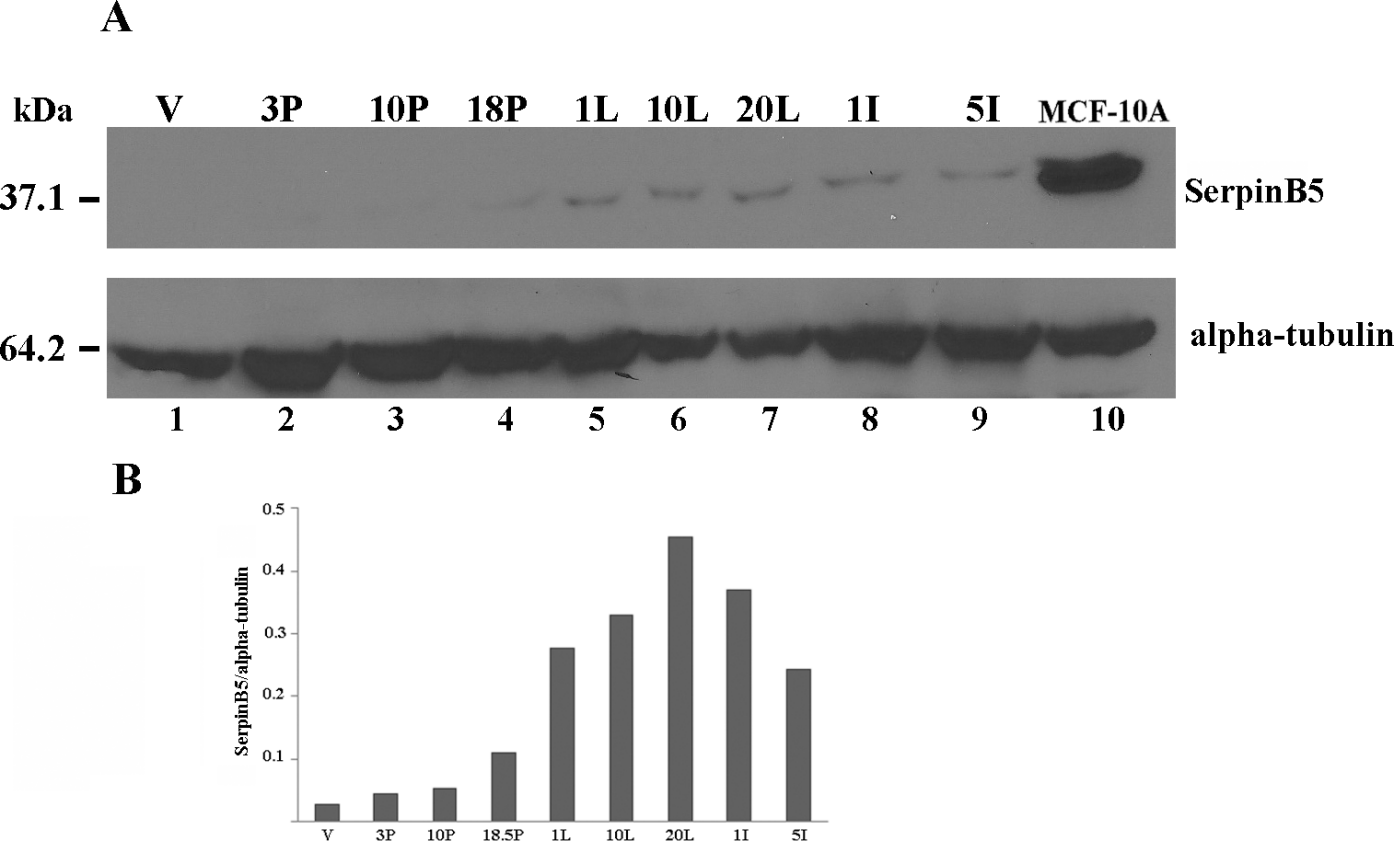
SerpinB5 expression is developmentally regulated in the mouse mammary gland. **A.** 100 μg of protein extracts from mammary glands isolated on days 3, 10, 18 of pregnancy (3P, 10P and 18P), on days 1, 10 and 20 of lactation (1L, 10L and 20L), on days 1 and 5 of involution (1I and 5I) and from virgin mice (V), were subjected to 12% SDS-PAGE, transferred to PVDF membrane and probed with mouse monoclonal anti-SerpinB5 (Millipore). 50 ug of MCF-10A protein extract was loaded as a positive control (lane 10). The membrane was reprobed with anti-alpha-tubulin for loading control (lower panel); **B.** The bar graph shows SerpinB5 levels normalized by alpha-tubulin. This result is representative of 3 independent experiments done with two different mouse strains.

SerpinB5 was initially described in human mammary epithelial cells as a single 42 kDa polypeptide chain with 89% homology with the mouse ortholog [1, 25]. Splicing variants have been described [26] but not characterized at protein level. We first determined SerpinB5 temporal expression and phosphorylation along the development of the mouse mammary gland. For this purpose, glands were isolated at different stages of development and SerpinB5 expression was analyzed in protein extracts by immunoblot (Fig 1A). SerpinB5 was undetectable in the adult virgin gland and in early pregnancy (lanes 1–2). A weak band was visible in late pregnancy (lane 4), which increases in lactation (lanes 5–7) and decreases slightly in involution (lanes 8–9). MCF-10A extract was used as a positive control (lane 10). This result is in good agreement with previous reports which analyzed SerpinB5 mRNA by northern blot [27] and protein levels by immunoblot [28], although the later did not look at SerpinB5 in the virgin, early pregnancy and late involution. Quantification of the bands indicates that SerpinB5 expression is highest during lactation (Fig 1B), reflecting previous observations [29]. This result suggests that SerpinB5 protein level is regulated during the development of the mouse mammary gland.

**Fig 2:**
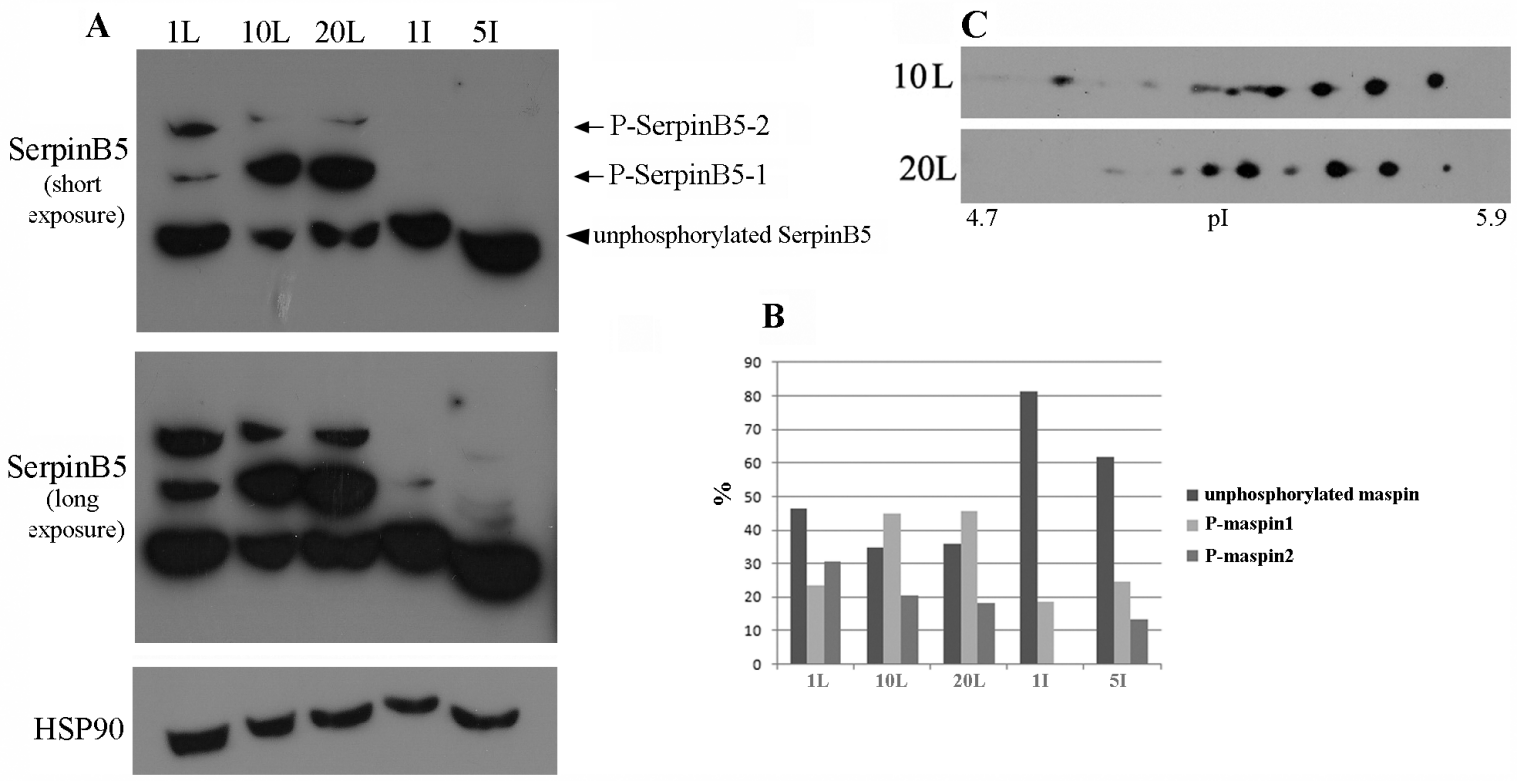
SerpinB5 phosphorylation in the lactating mammary gland. **A.** 100 ug of protein extracts from mammary gland isolated on days 1, 10 and 20 of lactation (1L, 10L and 20L), and on days 1 and 5 of involution (1I and 5I) were resolved by 8% Phos-Tag™ SDS-PAGE gel. Proteins were transferred to PVDF membrane and probed with rabbit anti-SerpinB5 (Sigma). Arrows indicate two phosphorylated SerpinB5 bands (P- SerpinB5-1 and P- SerpinB5-2) and arrowhead indicates unphosphorylated SerpinB5. Top and middle panels represent a short and a long X-ray film exposure, respectively. The membrane was reincubated with anti-HSP90 for a loading control; **B.** each of the three bands was quantified and expressed as percentage of the total levels of detected SerpinB5. This result is representative of two independent experiments; **C.** 2 mg of protein extracts of mammary gland isolated on days 10 and 20 of lactation were analyzed by 2D-SDS-PAGE using 17 cm pH 4.7–5.9 IPG strips followed by Western blot with anti-SerpinB5 (Santa Cruz). This result is representative of two independent experiments.

SerpinB5 post-translational modifications have been previously reported in different immortalized and transformed cell lines, including cysteine S-nitrosylation [30], phosphorylation on serine, threonine and tyrosine residues [20–22] and acetylation [31, 32]. However, the biological significance and the presence of these modifications *in vivo* and in different developmental stages have not been addressed. Since we have previously identified three different SerpinB5 phosphoforms in MCF-10A cells, a non-transformed human mammary epithelial cell line [21], we were interested in looking at SerpinB5 phosphorylation in the mammary gland. For this purpose, mammary glands from female mice at days 1, 10 and 20 of lactation and days 1 and 5 of involution were isolated, protein extracts were prepared and analyzed by Phos-Tag™ SDS-PAGE followed by immunoblot with anti-SerpinB5. We detected 3 different bands in this analysis: two of them correspond to phosphorylated SerpinB5 polypeptides, which we named P-SerpinB5-2 and P-SerpinB5-1 (Fig 2A, arrows). The third band has the highest electrophoretic mobility and corresponds to the unphosphorylated SerpinB5 (Fig 2A, arrowhead). Each of the bands was quantified and expressed as percentage of the total levels of SerpinB5 (Fig 2B). P-SerpinB5-1 and P-SerpinB5-2 bands likely contain more than one SerpinB5 phosphoisotypes which could not be resolved in a Phos-Tag™ SDS-PAGE gel. In order to determine how many SerpinB5 forms are present in the lactating gland, protein extracts of lactating glands isolated on days 10 and 20 were analyzed by 2D-SDS-PAGE on a 17 cm IPG strip containing a micro-range pH gradient (4.7–5.9), which allows the finest possible resolution [21]. SerpinB5 proteins were detected by immunoblot. We observed multiple spots in both samples which differ subtly in isoelectric point and intensity (Fig 2C). As addition of a phosphate group results in addition of a negative charge and a consequent decrease on the protein isoelectric point, these spots likely represent different SerpinB5 phosphoforms which differ slightly in their isoelectric point, and for this reason could not be separated by Phos-Tag™ SDS-PAGE. This result indicates the presence of multiple SerpinB5 phosphoforms in the mammary gland. In addition, SerpinB5 phosphorylation appears to be regulated during the development of the mouse mammary gland. We are currently investigating the identity of the kinases and phosphatases and the signaling pathways responsible for SerpinB5 phosphorylation in the mammary gland.

### EGFR signaling regulates SerpinB5 phosphorylation and nuclear localization in MCF-10A cells

**Fig 3:**
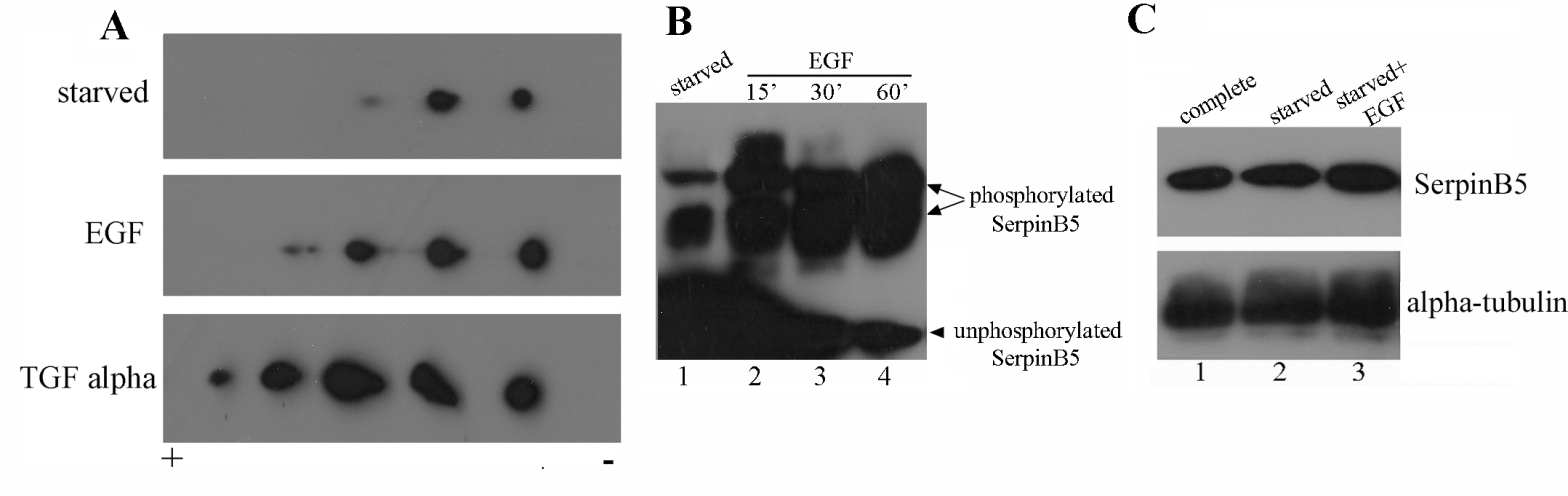
EGF and TGF alpha regulate SerpinB5 phosphorylation in MCF-10A cells. **A**. Starved MCF-10A cells were left untreated (upper panel) or treated with 20 ng/ml with EGF (middle panel) or TGF alpha (lower panel) for 20 minutes. 1.7 mg of protein extracts were analyzed by 2D-SDS-PAGE using 17 cm pH 4.7–5.9 IPG strips followed by Western blot with anti-SerpinB5 (BD Pharmigen); **B.** Starved MCF-10A cells were left untreated (lane 1) or treated with 20 ng/ml of EGF for the indicated periods of time (lanes 2–4). 50 ug of whole protein extracts were resolved by 8% Phos-Tag™ SDS-PAGE gel to separate the phosphorylated species. Proteins were transferred to PVDF membrane and probed with rabbit anti-SerpinB5 (Santa Cruz); **C.** MCF-10A cells were grown in complete medium (lane 1) or starved medium without (lane 2) or with 20 ng/ml of EGF for 15 min (lane 3). 50 ug of whole protein extracts were resolved by 12% SDS-PAGE, transferred to PVDF membrane and probed with anti-SerpinB5 (Millipore) or anti-alpha-tubulin for a loading control. This result is representative of two independent experiments.

In order to investigate the signaling pathways involved in SerpinB5 phosphorylation and the biological significance of this post-translational modification, we went back to the MCF-10A model, which expresses SerpinB5 endogenously and it is extensively employed as a model system to dissect different signaling pathways [33, 34]. Importantly, these cells exhibited many important features of non-transformed epithelial cells, markedly the dependence on hormones and growth factors for optimal proliferation and adhesion-dependent survival [35]. In our previous study we looked at SerpinB5 phosphorylation by 2D-SDS-PAGE followed by immunoblot in MCF-10A cells cultivated in the so called ‘complete medium’, i.e., medium containing insulin, EGF, cholera toxin, hydrocortisone and 5% horse serum [21]. In order to identify extracellular factors responsible for SerpinB5 phosphorylation, MCF-10A cells were cultivated in serum and soluble factors-free medium (i.e., starved cells) and whole protein extracts were analyzed by 2D-SDS-PAGE followed by immunoblot, as described in Fig 2C. SerpinB5 appears to be phosphorylated even in starved cells, as evidenced by the presence of three different spots (Fig 3A upper panel and 3B, lane 1). Since EGFR kinase domain phosphorylates recombinant SerpinB5 *in vitro* [22], we tested whether two high affinity EGFR ligands, EGF and TGF alpha, would modulate SerpinB5 phosphorylation. For this purpose, starved MCF-10A cells were treated with 20 ng/ml of EGF or TGF alpha for 20 minutes and whole cell extracts were analyzed similarly. Interestingly, both EGF and TGF alpha leads to an important increase in the number of SerpinB5 spots (Fig 3A, middle and lower panels), indicating that these EGFR ligands regulate SerpinB5 phosphorylation. To further confirm this finding, starved MCF-10A cells were treated with EGF for 15, 30 and 60 minutes and analyzed by Phos-Tag™ SDS-PAGE followed by immunoblot (Fig 3B lanes 2–4, arrows). EGF treatment results in increased SerpinB5 phosphorylation (Fig 3B lanes 2–4, arrows) with the expected decrease of unphosphorylated SerpinB5 (Fig 3B, arrowhead), without significant increase in SerpinB5 levels (Fig 3C).

We were then wondering about the origin of SerpinB5 phosphorylation in serum and growth factor-starved cells. Since SerpinB5 positively regulates cell adhesion and it is associated with Beta 1-integrin [24], we hypothesized SerpinB5 phosphorylation in starved cells could be due to signals emanating from the cell-substrate interaction. To test this possibility we used the previously described protocol for cell adhesion-signaling network analyses [36]. Starved MCF-10A cells were trypsinized and replated on self-deposited extracellular matrix or on control, BSA-coated surface for 1 hour at 37°C [24]. Although cells adhered and spread on matrix-coated surface much faster than on BSA (S1A Fig), 2D-SDS-PAGE followed by immunoblot indicates SerpinB5 phosphorylation levels did not differed substantially (S1B Fig), indicating that cell attachment is not responsible for SerpinB5 phosphorylation in starved cells.

**Fig 4:**
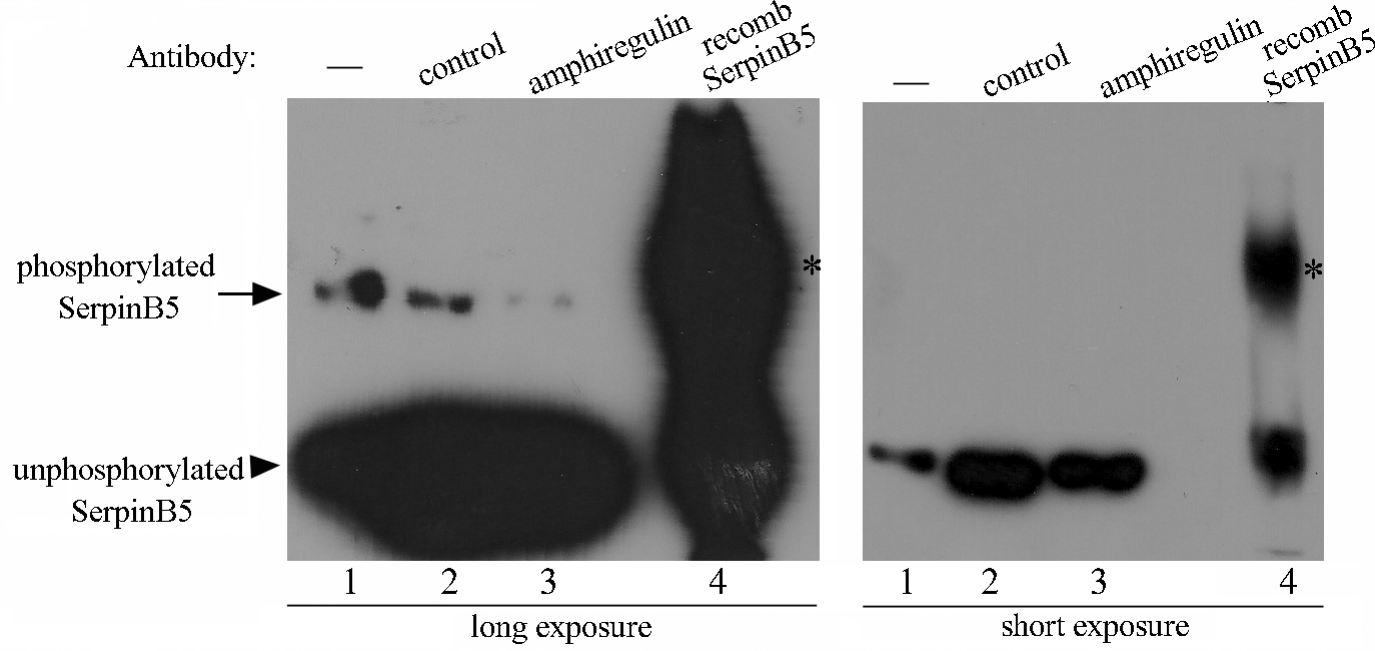
AREG is responsible for SerpinB5 phosphorylation in starved-MCF-10A cells. Starved MCF-10A cells (lane 1) were grown in medium containing either 1 ug/ml of goat IgG (lane 2) or goat anti-AREG-neutralizing antibody (lane 3) for 24 h. 50 ug whole protein extracts were resolved by 8% Phos-Tag™ SDS-PAGE gel. Recombinant SerpinB5 was used as unphosphorylated control (lane 4). Proteins were transferred to PVDF membrane and probed with anti-SerpinB5 (Sigma). A short exposure is shown on the right side; Asterisk indicates a spurious band. This result is representative of two independent experiments.

As MCF-10A cells secrete AREG (amphiregulin), another EGFR ligand [37], we reasoned SerpinB5 phosphorylation in starved cells could be a consequence of autocrine activation of EGFR by AREG. To test this hypothesis, MCF-10A cells were grown either in the presence of anti-AREG-neutralizing antibody or control antibody for 24 h and SerpinB5 phosphorylation was accessed by Phos-Tag™ SDS-PAGE. Interestingly, anti-AREG antibody resulted in a significant reduction of SerpinB5 phosphorylation (Fig 4, compare lanes 2 and 3), suggesting that autocrinally secreted AREG is indeed responsible for SerpinB5 phosphorylation in starved cells.

**Fig 5:**
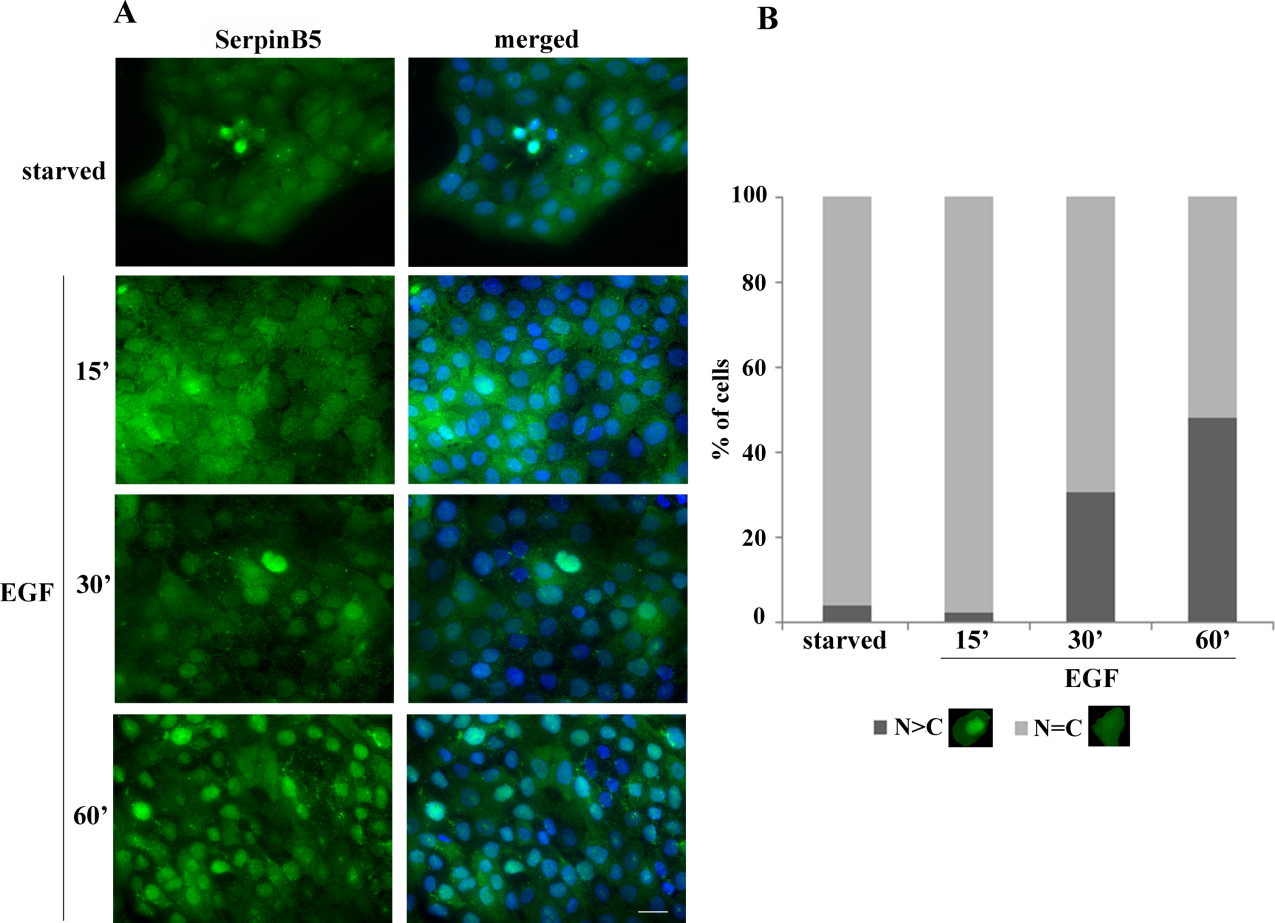
EGF treatment leads to SerpinB5 nuclear accumulation in MCF-10A cells. **A.** Starved MCF-10A cells were treated with 20 ng/ml of EGF for the indicated periods of time. Cells were fixed and processed for immunofluorescence with anti-SerpinB5 (Sigma). Nuclei were stained with DAPI. Bar, 50 μm; **B.** Cells were quantified based on the criteria shown under the graph. Approximately 130 cells from four random views were quantified. This result is representative of three independent experiments.

As we have previously observed a correlation between SerpinB5 phosphorylation and cytoplasmic localization in MCF-10A cells [21], we asked whether EGF treatment would result in changes in SerpinB5 subcellular localization as well. Starved MCF-10A cells were treated with EGF for different intervals and SerpinB5 localization was determined by immunofluorescence. SerpinB5 could be detected in the cytoplasm and in the nucleus in starved cells (Fig 5A). EGF treatment resulted in SerpinB5 accumulation in the nucleus in a time-dependent manner (Fig 5A and 5B). Since there is significant amount of SerpinB5 in the nucleus of starved cells, we tested whether AREG could be responsible for that. However, we did not detect any change in SerpinB5 nuclear localization in cells incubated with anti-AREG neutralizing antibody (S2 Fig). Altogether, these results indicate that at least three different EGFR ligands (EGF, TGF alpha and AREG) regulate SerpinB5 phosphorylation. Among them, only EGF can induce SerpinB5 nuclear translocation.

### SerpinB5 subcellular localization in the lactating mouse mammary gland

**Fig 6:**
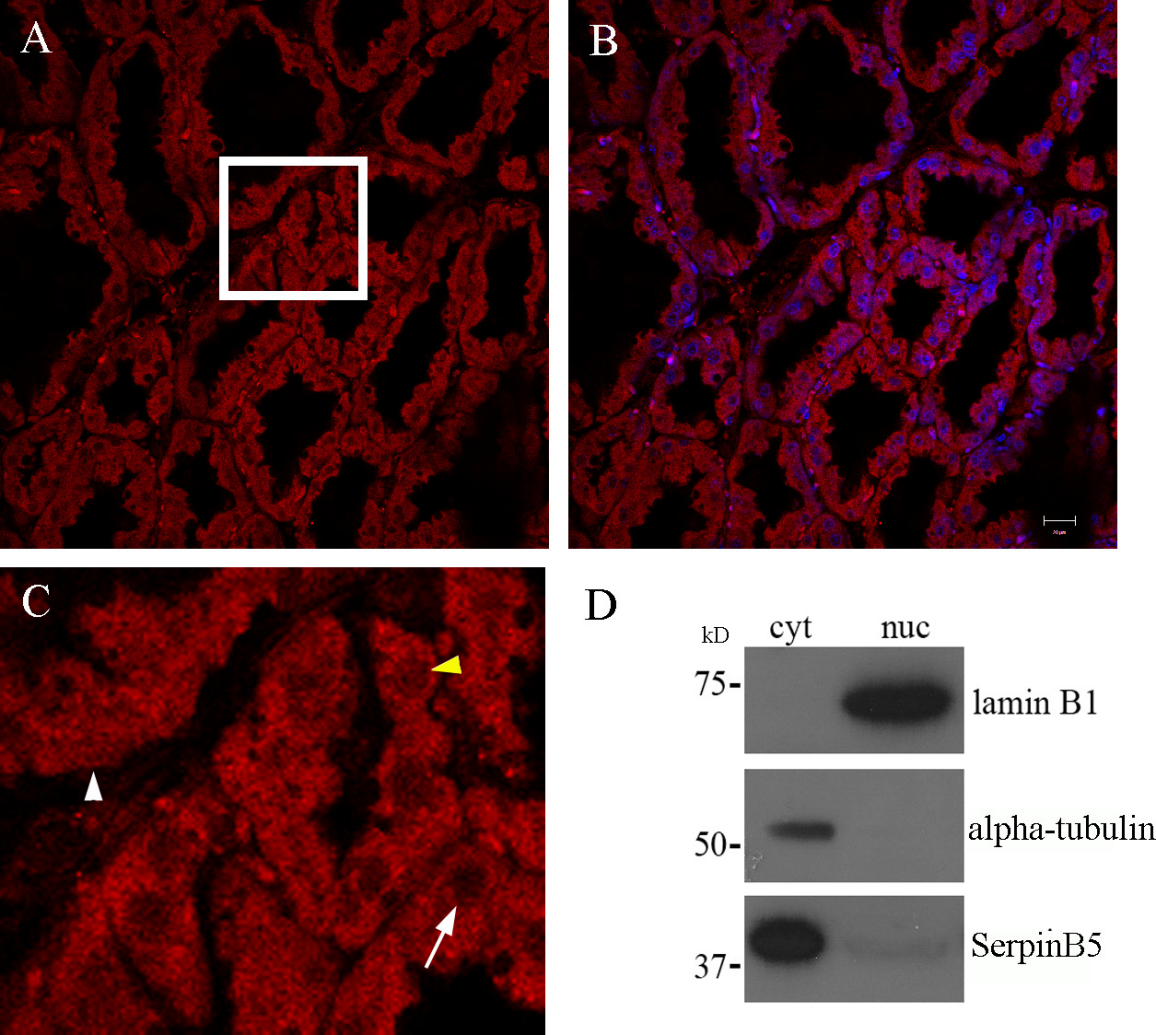
SerpinB5 subcellular localization in the mouse mammary gland. **A.** SerpinB5 immunofluorescence staining (Sigma) on paraffin section of a 10 day lactating gland; **B.** SerpinB5 and DAPI merge image; **C.** Representative area enlarged from boxed region indicating a SerpinB5-positive nucleus (white arrowhead), a partially-positive nucleus (yellow arrowhead) and a SerpinB5-negative nucleus (white arrow). **D.** 50 ug of nuclear (nuc) and cytoplasmic (cyt) protein fractions of a 10 day lactating mammary gland were subjected to 12% SDS-PAGE, transferred to PVDF membrane and probed with anti-SerpinB5 (Santa Cruz). Fractionation efficiency was monitored by reprobing the membrane with anti-Lamin B1 and anti-alpha-tubulin. Scale bar—20 μm.

Changes in SerpinB5 nucleocytoplasmic distribution were initially reported in several different tumor specimens, raising the hypothesis of a possible association between SerpinB5 subcellular localization and tumor progression. Attempts to correlate tumor progression/prognostic value and SerpinB5 nuclear or cytoplasmic localization resulted in great divergences and debates [13–19]. In normal human breast tissue, SerpinB5 is predominantly expressed by myoepithelial cells [38, 39], although weaker luminal epithelial staining has also been observed [1, 40]. In order to characterize SerpinB5 subcellular localization in a normal and physiological context, we looked at SerpinB5 by immunofluorescence and confocal microscopy during the developmental stage which most abundantly expresses it, the lactating mammary gland. Interestingly, whereas most, if not all luminal cells present SerpinB5 in the cytoplasm (Fig 6A and 6B), SerpinB5 nuclear staining presents differences which we attempted to classify as SerpinB5-filled nuclei, classified as such when nuclear boundaries could not be depicted (Fig 5C, white arrowhead); SerpinB5-negative nuclei (Fig 5C, white arrow) and SerpinB5 partially positive-nuclei, which is positive for SerpinB5 but nuclear boundaries could still be delineated (Fig 5C, yellow arrowhead). To confirm this finding, SerpinB5 expression was analyzed by immunoblot in the nuclear and cytoplasmic fractions of the lactating mammary gland. In agreement with the image analysis, SerpinB5 was more abundant in the cytoplasmic fraction, but could also be detected in the nucleus (Fig 6D). Very similar results were observed when the same analysis was done with the involuting mammary glands isolated on days 1 and 5 (S3 Fig). These results indicate that SerpinB5 is predominantly in the cytoplasm and its subcellular localization does not change significantly during mammary gland development. In addition, nuclear SerpinB5 levels appear to differ among different cells.

## Discussion

In this study we determined maspin/SerpinB5 protein expression, phosphorylation and subcellular localization during the development of the mouse mammary gland and investigated the signaling pathways involved in SerpinB5 phosphorylation and nuclear translocation in MCF-10A cell culture. In the mammary gland, SerpinB5 protein was detected during lactation and involution, whereas phosphorylation clearly predominates in lactation. These observations suggest SerpinB5 expression and phosphorylation are developmentally regulated processes and therefore may be under hormonal control. By phos-tag gel analysis followed by immunoblot, 2 phosphoforms, referred as P-SerpinB5-1 and P-SerpinB5-2, were detected (Fig 2A). Biochemically, these forms differ in the amount of phosphate groups covalently bound to the SerpinB5 peptide chain; thus P-SerpinB5-2 has more phosphate groups than P-SerpinB5-1. The biological significance of these SerpinB5 phosphoforms, however, is not known. Interestingly, P- SerpinB5-1 levels increase importantly throughout lactation while P-SerpinB5-2 levels display a rather modest decrease. The numerous SerpinB5 phosphoforms identified by 2D-SDS-PAGE (Fig 2C) further supports a model in which developmentally regulated kinases and phosphatases keep SerpinB5 phosphorylation under a tight control during lactation. The prevalence of phospho-SerpinB5 during lactation suggests that these forms may have a particular and restricted role during this developmental period. The sudden decrease in SerpinB5 phosphorylation at the onset of involution further supports this hypothesis. *SerpinB5* target expression under the control of the whey acidic protein promoter, a milk protein gene, resulted in decreased milk protein expression, increase in apoptosis and impaired lobuloalveolar development [27]. In addition, *SerpinB5* was detected among the top 10 genes uniquely expressed during the lactating mammary gland [41]. Altogether, these data indicate that SerpinB5 plays an important, yet poorly understood role during the lactation stage of the mammary gland. As SerpinB5 protein is also detected throughout involution, it may also play a role in mammary gland remodeling and cell death, as has been previously suggested [29]. In line with this observation, SerpinB5 sensitizes cells to cell death [42, 43] and it is a cathepsin D binding partner [44], an instigator of mammary gland involution [45]. We found that EGF and TGF alpha regulates SerpinB5 phosphorylation and autocrinally secreted AREG, and not an adhesion-induced signal, is responsible for SerpinB5 phosphorylation in starving MCF-10A cells. Of note, whereas EGF treatment is followed by SerpinB5 nuclear translocation, anti-AREG neutralizing antibody efficiently inhibits SerpinB5 phosphorylation without interfering with its subcellular localization (S2 Fig). Different EGFR agonists lead to different cell responses even on the same cell type [46]. This difference has been clearly demonstrated for EGF and AREG in the mammary gland [47, 48]. The effect of TGF alpha on SerpinB5 subcellular localization has not been analyzed in this study. Based on our observation for EGF and AREG and on what is currently known about EGFR signaling, TGF alpha-induced SerpinB5 phosphorylation may regulate different, yet unknown function of this protein in the cell. The observation that SerpinB5 is located in the nucleus even in starved cells indicate that SerpinB5 nuclear localization is regulated by at least two different mechanisms–one non-regulated mechanism which is already present in resting cells and another which depends on EGF pathway activation. As SerpinB5 molecular weight (42 kDa) is close to the size limit for passive diffusion [49], it is theoretically possible that SerpinB5 nuclear translocation occurs via a passive mechanism. However, a simple SerpinB5 cDNA transfection in TM40D mammary tumor cells resulted in SerpinB5 in the cytoplasm only [50], suggesting that SerpinB5 requires an active mechanism to translocate to the nucleus. Interestingly, SerpinB5-GFP expression (which is about 67 kDa) was restricted to the cytoplasm [15, 51] or found in both compartments (cytoplasm and nucleus) [52] depending on the cell type. One possible explanation for this difference is the presence of different ratios of SerpinB5-GFP to endogenous cytoplasmic anchoring proteins in these cell lines. If anchoring proteins are limited, GFP-SerpinB5 may translocate to the nucleus, as has been previously observed for ERK2 [53]. It is also possible that SerpinB5 subcellular localization is actively regulated by different signaling pathways, as we observed here for EGF in MCF-10A cells. As cell lines differ in their signaling pathways, this difference could account for the differences in SerpinB5 subcellular localization in these cell lines. A number of different proteins have their nuclear localization regulated by EGF, including its own receptor, EGFR [54], ERK2 [55], pyruvate kinase M2 [56] and Stat5 [57]. EGF can induce protein nuclear translocation via diverse mechanisms. For example, EGF signaling does not directly act on ERK2 nuclear translocation signal, but rather releases it from cytoplasmic anchors [53], which is essential for ERK2 nuclear translocation. For PMK2, EGF induces PMK2 phosphorylation and acetylation, which are both essential for PMK2 nuclear translocation [58, 59]. The cytoplasmic levels of SerpinB5 remained high after EGF treatment, suggesting that only a fraction of cellular SerpinB5 translocates to the nucleus. In the nucleus, SerpinB5 likely regulates gene expression, as it is physically associated with histone deacetylase 1 (HDAC1) [60] and interferon regulatory factor 6 (IRF6), a transcription factor which regulates cell cycle arrest and quiescence during cell differentiation [61]. It has been hypothesized that IRF6 and SerpinB5 cooperate to establish cell cycle arrest and terminal differentiation in mammary epithelial cells during lactation [28]. How the interaction between IRF6 and SerpinB5 ultimately regulates cell differentiation is still not clear. Bailey *et al*. observed that IRF6 phosphorylation leads to its ubiquitination and degradation by the proteasome. Interestingly, SerpinB5 binds preferentially to the phosphorylated IRF6. They hypothesized that SerpinB5 may act as an IRF6 anchor, preventing IRF6 degradation and perhaps nuclear translocation [29]. In MCF-10A cells, EGF alone is not able to promote IRF6 phosphorylation and does not induce entry in the cell cycle. A recent study demonstrated that EGF (and not others EGFR ligands) is specifically upregulated during lactation in the mouse mammary gland and it is essential for alveolar cell survival during this particular developmental stage [62], raising the interesting hypothesis of a role of SerpinB5 in the EGF-dependent function *in vivo*. In this context, it is possible that EGF-induced SerpinB5 translocation results in IRF6 release, which would then promote cell cycle arrest for mammary epithelial cell differentiation. We are currently investigating if EGF regulates SerpinB5 in the mouse mammary gland. While the results presented here suggest that EGF-induced SerpinB5 phosphorylation may regulate SerpinB5 nuclear localization, our previous results showed SerpinB5 accumulation in the cytoplasm in sodium pervanadate-treated cells, a tyrosine phosphatase inhibitor. Altogether, these data indicate that two different SerpinB5 fractions have their subcellular localization regulated by phosphorylation. Whether these events occur independently or are associated in the cells remains to be established. The biological significance of the different patterns of SerpinB5 nuclear staining in the lactating mammary gland is not clear. It will be interesting to determine how SerpinB5 nuclear status relates to the expression of estrogen and progesterone receptors, as these steroid hormones orchestrate mammary epithelial proliferation and morphogenesis [63]. The predominance of SerpinB5 in the cytoplasm of mouse mammary epithelial cells contrasts with studies which found a positive correlation between SerpinB5 cytoplasmic localization and poor breast cancer outcome [15, 17], emphasizing the importance of understanding SerpinB5 function in a non-transformed context. We are presently identifying SerpinB5 phosphorylated residues by mass spectrometry and dissecting the components of the signaling pathway involved in SerpinB5 phosphorylation and subcellular localization, as well as the translocation machinery involved. Future studies will aim at investigating the role of SerpinB5 in mammary gland development and how it is altered during mammary tumorigenesis.

## Conclusions

The present study reveals for the first time the status of SerpinB5, a tumor suppressor gene, during the development of the mammary gland regarding its phosphorylation and subcellular localization. We identified numerous SerpinB5 phosphoforms which are developmentally regulated. SerpinB5 is abundantly expressed by luminal cells and is predominantly located in the cytoplasm. In contrast, there are different patterns of SerpinB5 nuclear staining, suggesting that the levels of this protein in the nucleus is under tight control. In addition, we revealed that EGF signaling regulates these two processes in a non-transformed *in vitro* model. These results provide important insights towards understanding the role of SerpinB5 in the mammary gland biology, which will ultimately be able to reconcile the important divergences in the field regarding the role of SerpinB5 as a breast tumor suppressor and help design more effective therapeutic and prognostic tools.

## Supporting Information

S1 Fig Cell-substrate interaction does not affect SerpinB5 phosphorylation. **A.** Starved MCF-10A cells were trypsinized and replated on BSA (upper panel) or on MCF10A-self-deposited extracellular matrix (lower panel) and incubated for 1 h at 37°C [24]. **B.** Protein extracts were prepared and analyzed by 2D-SDS-PAGE using 17 cm pH 4.7–5.9 IPG strips followed by Western blot with anti-SerpinB5 (Millipore). This result is representative of two independent experiments.
(TIF)

S2 Fig AREG does not regulate SerpinB5 nuclear accumulation in starved MCF-10A cells. Starved MCF-10A cells were grown in medium containing either 1 ug/ml of goat IgG or goat anti-AREG-neutralizing antibody, as indicated on the Fig Cells were fixed and processed for immunofluorescence with anti-SerpinB5 (Sigma). Nuclei were stained with DAPI.
(TIF)

S3 Fig SerpinB5 subcellular localization in the involuting mammary gland. 50 ug of nuclear (nuc) and cytoplasmic (cyt) protein fractions of a 1 day (**A**) or 5 day (**B**) involuting mammary gland were subjected to 12% SDS-PAGE, transferred to PVDF membrane and probed with anti-SerpinB5 (Santa Cruz). Fractionation efficiency was monitored by reprobing the membrane with anti-Lamin B1 and anti-alpha-tubulin.
(TIF)
