# Supplementary figures and images for: EGFR Signaling Regulates Maspin/SerpinB5 Phosphorylation and Nuclear Localization in Mammary Epithelial Cells
Source: PLoS One. 2016 Jul 22;11(7):e0159856. doi: 10.1371/journal.pone.0159856 (PMC4957797; doi:10.1371/journal.pone.0159856)

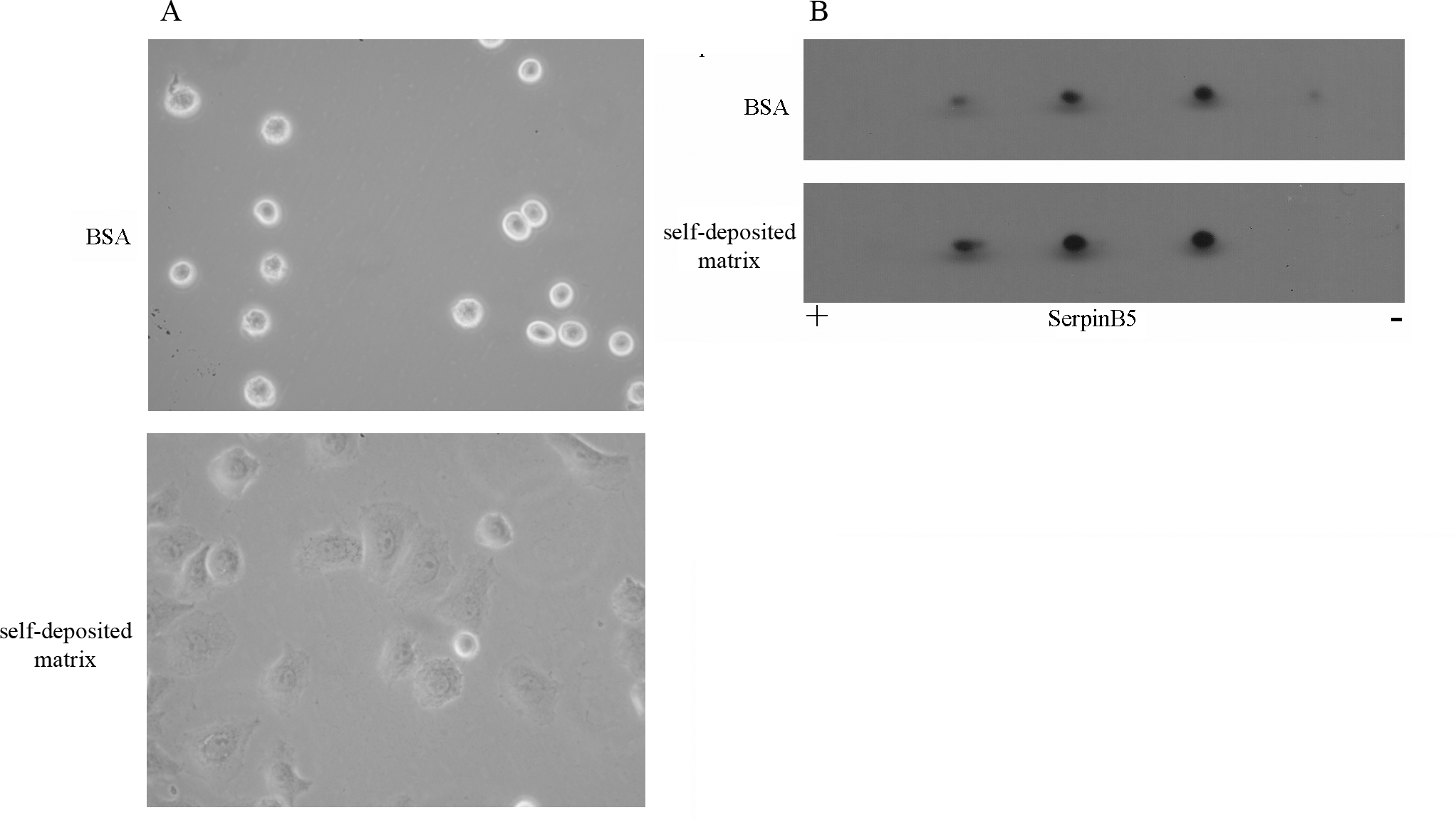

Supplement: S1 Fig — A. Starved MCF-10A cells were trypsinized and replated on BSA (upper panel) or on MCF10A-self-deposited extracellular matrix (lower panel) and incubated for 1 h at 37°C [24]. B. Protein extracts were prepared and analyzed by 2D-SDS-PAGE using 17 cm pH 4.7–5.9 IPG strips followed by Western blot with anti-SerpinB5 (Millipore). This result is representative of two independent experiments. (TIF) [file pone.0159856.s001.tif]

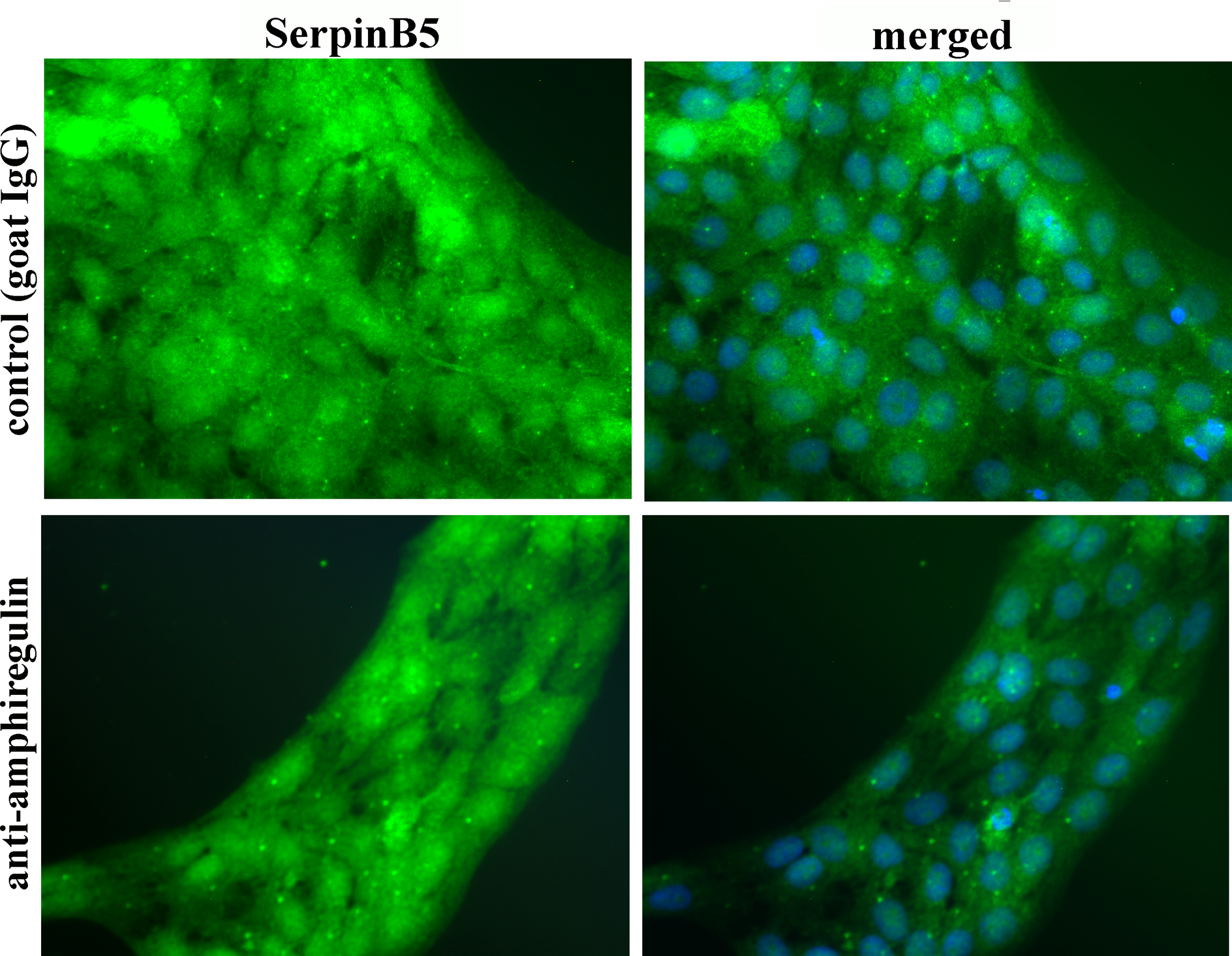

Supplement: S2 Fig — Starved MCF-10A cells were grown in medium containing either 1 ug/ml of goat IgG or goat anti-AREG-neutralizing antibody, as indicated on the Fig Cells were fixed and processed for immunofluorescence with anti-SerpinB5 (Sigma). Nuclei were stained with DAPI. (TIF) [file pone.0159856.s002.tif]

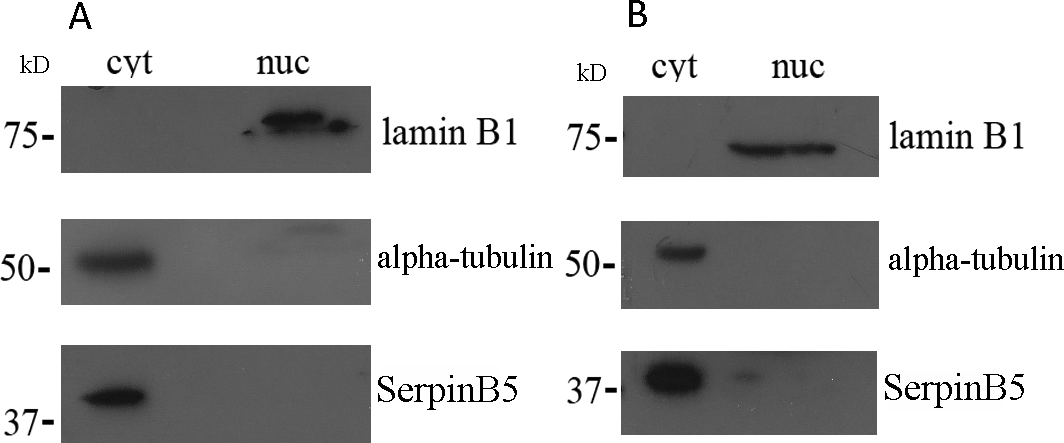

Supplement: S3 Fig — 50 ug of nuclear (nuc) and cytoplasmic (cyt) protein fractions of a 1 day (A) or 5 day (B) involuting mammary gland were subjected to 12% SDS-PAGE, transferred to PVDF membrane and probed with anti-SerpinB5 (Santa Cruz). Fractionation efficiency was monitored by reprobing the membrane with anti-Lamin B1 and anti-alpha-tubulin. (TIF) [file pone.0159856.s003.tif]
